# The occurrence of *Alaria alata* mesocercariae in wild boars (*Sus scrofa*) in north-eastern Poland

**DOI:** 10.1016/j.ijppaw.2020.04.006

**Published:** 2020-04-20

**Authors:** Natalia Strokowska, Marek Nowicki, Daniel Klich, Zbigniew Bełkot, Jan Wiśniewski, Anna Didkowska, Piotr Chyla, Krzysztof Anusz

**Affiliations:** a Department of Food Hygiene and Public Health Protection, Institute of Veterinary Medicine, Warsaw University of Life Sciences, Nowoursynowska 159, 02-776, Warsaw, Poland; b Department of Animal Genetics and Conservation, Institute of Animal Science, Warsaw University of Life Sciences, Ciszewskiego 8, 02-786, Warsaw, Poland; c Department of Food Hygiene of Animal Origin, Faculty of Veterinary Medicine, University of Life Sciences in Lublin, Akademicka 12, 20-950, Lublin, Poland

**Keywords:** *Alaria alata*, Mesocercariae, *Distomum musculorum suis*, Wild boar, AMT

## Abstract

Various species of mammals, including humans and wild boars, can serve as paratenic hosts of *Alaria alata* mesocercariae – *Distomum musculorum suis* (DMS). Cases of DMS can be reliably detected by the recent introduction of the *A. alata* mesocercariae migration technique (AMT). The aim of this study is to present current data on the occurrence of DMS in wild boars in north-eastern Poland, and to compare the findings with those obtained in other European countries. *A. alata* was identified in 98 of 221 (44.3%) tissue samples of wild boar taken from five provinces in north-eastern Poland during the hunting seasons 2015–2016 and 2016–2017 based on AMT analysis. Positive results were found in all studied regions, but the percentage of infected individuals ranged from 26.3% in the Pomorskie province to 65.5% in the Warmińsko-Mazurskie province. The mean number of larvae exceeded seven larvae per 30 g sample for three provinces: Pomorskie, Mazowieckie and Lubelskie. In turn, lower values were found in the Warmińsko-Mazurskie province (3.3 larvae per 30 g), and the lowest in the Kujawsko-Pomorskie province (1.8 larvae per 30 g). The occurrence and intensity of *A. alata* infestation in wild boars was found to depend on the environment in which they live. Neither the sex or the age of the wild boar appeared to influence the occurrence nor the intensity of infestation.

## Highlights

• High percentage (44.3) of *Alaria alata* in samples of wild boars in Poland.
• High prevalence of *A. alata* infestation in wild boars compared to other reports.
• Sex does not affect the risk or intensity of infestation by *A. alata* in wild boars.
• Age does not affect the risk or intensity of *A. alata* infestation in wild boars.
• *A. alata* poses a potential risk for wild boar consumers in Poland.

## Graphical abstract

**Figure:**
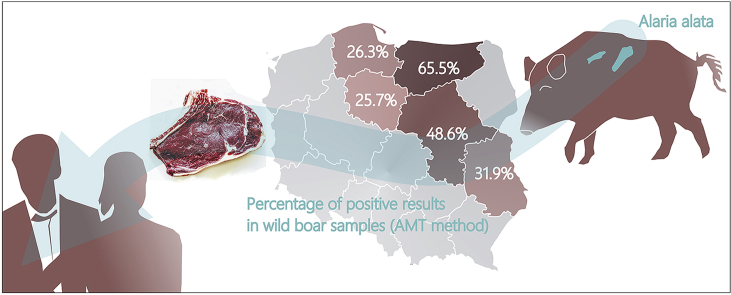

## 1 Introduction

The trematode *A. alata* (Goeze, 1782) and its mesocercariae – *Distomum musculorum suis* (DMS) (Duncker, 1896) are known to parasitize various carnivorous animals such as canines, felines or mustelids (Takeuchi-Storm et al., 2015a, Takeuchi-Storm et al., 2015b; Bindke et al., 2019; Nugaraitė et al., 2019; Miljević et al., 2019; Ozoliņa et al., 2020). Their life cycle includes two intermediate hosts: a freshwater snail and an amphibian, such as a frog. In addition, many species of omnivorous and carnivorous mammals can also act as paratenic hosts, i.e. they harbor an immature parasite which does not develop: they are not essential for the development cycle of the parasite (Schimalov, 2017; Rentería-Solís et al., 2013, 2018). Wild boars are common paratenic hosts because of their diverse feeding habits: their diet comprises various plants, roots, insects, molluscs, amphibians, small mammals and sometimes carrion.

In Poland, wild boar is the most commonly-hunted game. The Polish Central Statistical Office reports that the population grew constantly from about 118,000 to 285,000 individuals over the period 2000–2014. However, wild boar shooting was expanded in 2014, due to the occurrence of African swine fever (ASF), and as a result, the population has gradually decreased, reaching 215,000 individuals in 2017. Current annual quotas are about 200,000 animals. Although wild boar meat consumption remains popular in Poland, to protect public health, it needs to be thoroughly tested for *Trichinella spiralis* and other pathogens*.* One such agent that has received recent attention is *Alaria alata.*

Current EU legislation, i.e. in accordance with Commission Regulation (EC) No 2075/2005 of 5 December 2005, specifies that wild boar meat should be examined for the presence of *T. spiralis* using the TRM (*Trichinella* reference method) - magnetic stirrer method for pooled sample digestion. Although the presence of *A. alata* mesocercariae (DMS) can be detected incidentally during this procedure, a more sensitive method for directly detecting DMS is the *Alaria* mesocercariae migration technique (AMT) (Riehn et al., 2010). AMT is also a simple, fast and low-cost method that can not only be performed on muscle samples, as in the *Trichinella* test, but also on other tissue samples (Riehn et al., 2013). Hence, the AMT method has been recommended for DMS detection by a number of laboratories, including the Chief Veterinary Officer of Poland and National Veterinary Research Institute in Puławy.

*Alaria* trematode infestation is known to cause alariosis, a zoonosis that is gaining importance and is classified as a “re-emerging disease” in Europe (Möhl et al., 2009). Cases caused by *Alaria americana* have been reported in humans, consisting of respiratory symptoms, dermal and ocular infections (Beaver et al., 1977; McDonald et al., 1994; Kramer et al., 1996). Although no human cases of alariosis caused by *A. alata* have been detected so far, *A. alata* is closely related to *Alaria americana,* and is classified as a zoonotic agent by various organizations, including the Swiss Agency for the Environment, Forests and Landscape (SAEFL).

*A. alata* is a species of *Alaria* which naturally occurs in Europe. Its adult and mesoceraria forms have been found in many countries in Europe: Greece (Papadopoulos et al., 1997), Ireland (Wolfe et al., 2001; Murphy et al., 2012), Belarus (Shimalov and Shimalov, 2003), Austria (Sailer et al., 2012), France (Portier et al., 2012), the Czech Republic (Paulsen et al., 2013), Germany (Rentería-Solís et al., 2013), Hungary (Széll et al., 2013), Bulgaria (Riehn et al., 2014), Latvia (Ozoliņa et al., 2018) and Serbia (Gavrilović et al., 2019), as well as Poland (Wójcik et al., 2001; Górski et al., 2006; Popiołek et al., 2007; Szafrańska et al., 2010; Karamon et al., 2018; Rentería-Solís et al., 2018). The aim of the present study is to present current data on the occurrence of DMS in wild boars in selected regions of Poland, to compare these findings with those in other European countries and to determine the relationship between the occurrence of DMS in wild boars and their age, sex and place of residence.

## 2 Materials and methods

### 2.1 Types of samples

**Fig. 1:**
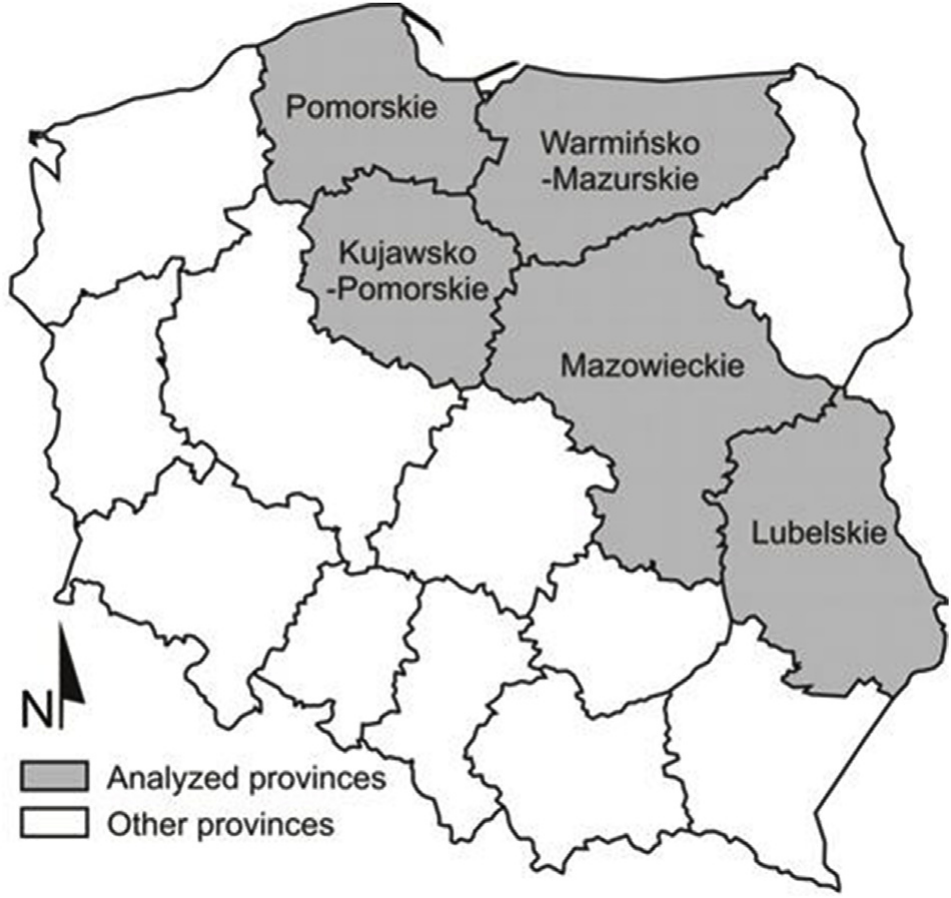
Locations of collected samples from wild boar in Poland.

Samples were taken from 221 wild boars hunted in Kujawsko-Pomorskie, Lubelskie, Mazowieckie, Pomorskie and Warmińsko-Mazurskie provinces between October and September in the 2015–2016 and 2016–2017 hunting seasons (Fig. 1). The choice of hunting ground depended on the preferences of local hunters. The sample size ranged from 39.12 g to 248.34 g (average = 119.02, SD = 55.37, median = 104.12) and consisted of muscle tissue from the diaphragm, peridiaphragmatic adipose tissue and connective tissue from the central tendon of the diaphragm. Until analysis, the samples were stored at 2–4 °C. The date and location of the hunt, as well as the sex, age and weight of the wild boar were documented.

### 2.2 Examination method

Samples containing muscles, adipose tissue and connective tissue were tested with the AMT method as described previously (Riehn et al., 2010). The parasites were identified by their characteristic movements and morphological features. The samples were tested within seven days of acquisition.

### 2.3 Statistical analysis of data

Statistical analyses were performed using SPSS software (version 24.0, IBM Corporation, Armonk, NY). The presence of *A. alata* in the wild boar was determined using a generalized linear model for binary data with a log-link function. The presence of *A. alata* in the sample was determined as a response variable and the location (province) and age of the wild boar (young/adult) were determined as factors. Positive results concerning the detection of *A. alata* in the tested samples were analyzed with a generalized linear model, in which the number of mesocercariae in the standardized sample was a response variable, and the location (province) and age of the wild boar (young/adult) were constant factors.

A negative binomial distribution with log-link function was used after comparison with other distributions for count data using the value of Akaike information criterion. In both models, individuals under two years of age (piglets and subadults), weighing up to 40 kg were treated as juveniles, and individuals aged two years and over were treated as adults. Different types of model, including interactions, were compared by backward elimination to obtain the best-fitting models based on the Akaike information criterion. Statistically significant variables in the models were evaluated in pairwise comparisons with the least significant difference (LSD) test.

## 3 Results

The presence of *A. alata* mesocercariae was confirmed in 98 of the 221 (44.3%) collected samples. Although positive results were found in all provinces, the percentage of infected individuals ranged from 26.3% in the Pomorskie province to 65.5% in the Warmińsko-Mazurskie province (Table 1). The best model of *A. alata* presence was highly statistically significant (χ2 = 20.09; df = 4; P = 0.000). The only statistically significant factor was the location, i.e. the province (Wald χ2 = 18.68; df = 4; P = 0.001); neither the age of the individuals nor the age * province interaction were statistically insignificant, and were hence excluded during AIC backward elimination. The Warmińsko-Mazurskie province demonstrated the highest probability of wild boar infection; this location also differed significantly from other provinces, except for Mazowieckie province, with 48.6% of positive results. The Mazowieckie province itself was found to have the second highest number of positive results and to differ significantly from the next highest province, Kujawsko-Pomorskie, with 26.7% of positive results. No other statistically significant differences were found (Table 1).

**Table 1:** The effects of location (province) on the presence of *A. alata* in wild boar samples as a pairwise comparison in the least significant difference test (for the entire model: *χ2 = 20.09; df=4; P=0.000*; for intercept: *Wald χ2 = 7.26; df=1; P = 0.007,* for location as a factor: *Wald χ2 = 18.68; df=4; P = 0.001*), * statistically significant.

Province	N	Positive results [%]	Significance level (*P value*) in LSD test				
Pomorskie (POM)	19	26.3					
Warmińsko-Mazurskie (W-M)	55	65.5	0.001*				
Kujawsko-Pomorskie (K–P)	30	26.7	0.978	0.000*			
Mazowieckie (MAZ)	70	48.6	0.058	0.054	0.029*		
Lubelskie (LUB)	47	31.9	0.646	0.000*	0.619	0.066	
province (pairwise comparison)			(POM)	(W-M)	(K–P)	(MAZ)	(LUB)

The best model for the count of AM in a standardized wild boar sample included both factors (location and age) and the interaction between location and age (Table 2). The mean number of larvae varied significantly with regard to location (Wald χ2 = 10.76; df = 4; P = 0.029). Although the Akaike criterion did not exclude age as a factor in the model, no significant differences were observed between young and adult wild boars. Significant differences were observed in the interaction of location * age (Wald χ2 = 8.34; df = 3; P = 0.039).

**Table 2:** Statistical summary of generalized linear model of *Alaria alata* mesocercaria numbers in a standardized sample and predictors: location (province), age (young/adult) and interaction location*age; for the entire model: *χ2 = 27.65; df=8; P = 0.001*, N = 96 (*statistically significant).

Source	*Wald χ2*	*Df*	*P*
Intercept	59.55	1	0.000*
Location	10.76	4	0.029*
Age	0.04	1	0.835
Location*age	8.34	3	0.039*

Statistically similar mean AM values were observed among three provinces, *viz.* Pomorskie, Mazowieckie and Lubelskie, all of which exceeded seven cases per 30 g sample (Table 3). The Mazowieckie and Lubelskie provinces also demonstrated the highest number of mesocercariae in selected samples, respectively 79 and 54 specimens per sample. The lowest mean value (1.8 per 30 g sample) was observed in the Kujawsko-Pomorskie; this value differed significantly from those of the Mazowieckie and Lubelskie provinces (P = 0.003 and P = 0.020, respectively). Moderate mean values were observed in the Warmińsko-Mazurskie province (3.3 per 30 g of the sample); these differed significantly from those observed in the Mazowieckie province (P = 0.016). No other significant differences were found, mainly due to the small number of observations or a high standard error in the province (Table 3). The differences between the indicated provinces concerned mainly young wild boars in the Mazowieckie province; these were found to have a mean value of 11.5 *A. alata* mesocercariae per 30 g sample, which significantly differed from the values obtained for almost all young and adult wild boars in the remaining provinces (P < 0.005).

**Table 3:** The effect of location (province) on *A. alata* mesocercaria numbers in wild boar samples as a pairwise comparison in the least significant difference test (*Wald χ2=18.68; df=4; P = 0.001)*, * statistically significant.

Province	N	Mean ± SE	Min-Max	Significance level (*P value*) in LSD test				
Pomorskie (POM)	5	7.20 ± 3.44	1–17					
Warmińsko-Mazurskie (W-M)	36	3.29 ± 0.90	1–13	0.270				
Kujawsko-Pomorskie (K–P)	6	1.79 ± 0.48	1–3	0.137	0.320			
Mazowieckie (MAZ)	34	7.12 ± 1.32	1–74	0.983	0.016*	0.003*		
Lubelskie (LUB)	15	7.67 ± 2.23	1–59	0.908	0.068	0.020*	0.831	
Province (pairwise comparison)				(POM)	(W-M)	(K–P)	(MAZ)	(LUB)

## 4 Discussion

In Poland, most studies on the detection of *A. alata* in wild boar meat discuss the detection of DMS during official examination for the presence of *Trichinella* spp. based on the TRM method (Wójcik et al., 2002; Michalski and Wiszniewska-Laszczych, 2016). For example, Michalski and Wiszniewska-Laszczych (2016) report the accidental detection of DMS in five of 83 (6.02%) samples taken from hunted wild boars from Poland, which was a similar result to those observed in other countries (Jaksic et al., 2002; Gazzonis et al., 2018; Gavrilović et al., 2019). A much higher prevalence was observed in the present study; however, this may be due to the fact that the AMT method has greater sensitivity to *A. alata* mesocercariae and can detect infestations at very low intensities (Riehn et al., 2010), unlike the magnetic stirrer method for pooled sample digestion. The higher specificity of the AMT method for identifying the presence of *Trichinella* spp. may be associated with the fact that *A. alata* mesocercariae are larger than *T. spiralis* larvae and tend to be retained on the sieves used in the TRM approach (Ozoliņa and Deksne, 2017).

Our results indicate that 103 of the 221 studied samples (44.3%) were positive. This value is considerably higher than previous findings regarding the prevalence of *A. alata* in wild boars; however, higher values were confirmed in Lithuania (46/60, 76.7%) also using the AMT (Ozoliņa and Deksne, 2017). These values are well above the range described in previous reports from Austria (Sailer et al., 2012; Riehn et al., 2012) and the Czech Republic (Paulsen et al., 2013). Differences in the prevalence and intensity of *A. alata* infestation have also been observed in intermediate hosts; for example, the number of larvae ranged from several to 314 per individual among Pelophylax frogs (Patrelle et al., 2015; Voelkel et al., 2018).

The high prevalence noted in Poland may be connected with environmental conditions. The region from which the samples were taken is rich in lakes, marshes and rivers, which makes it an ideal habitat for intermediate hosts, such as water snails and frogs, as well as the wild boars. Further research should also include other provinces.

Few significant differences were found between the provinces with regard to mean number of *A. alata* mesocercariae per sample. The number of samples with high dispersion of results was limited.

*Alaria* spp. mesocercariae in game can pose risk for humans (Fried and Abruzzi, 2010). In our opinion, it seems reasonable to consider *A. alata* as an agent endangering human health. Therefore, it is recommended that wild boar meat is frozen or processed at high temperatures before consumption in order to inactivate and kill mesocercariae (González-Fuentes et al., 2014). This would appear to be a particularly important measure in Poland, considering the high percentage of positive results for DMS in samples. In addition, it would be advisable to re-evaluate the regulations regarding mandatory inspection for *A. alata*.

## 5 Conclusions

The occurrence of *A. alata* in wild boars depends on the environment in which they live. The percentage of infected individuals ranged from 26.3% to 65.5% according to province. In contrast to previous reports, our findings reveal a wide prevalence of *A. alata* infestation among wild boars in Poland. The occurrence and intensity did not depend on the sex of the wild boar. Similarly, age did not appear to influence the occurrence of *A. alata* in wild boars or intensity of the infestation, assuming a division between juveniles, aged up to two years old, and adults, aged over two years; however, juveniles from Mazowieckie province presented higher intensity than almost all individuals (juvenile or adult) in other provinces. The presence of the *A. alata* infestation is not a marginal trend in Poland and there is a need for further research in this area.

## Declaration of competing interest

In behalf of all authors I declare that there is now conflict of interests.
